# Improving the Health Benefits of Snap Bean: Genome-Wide Association Studies of Total Phenolic Content

**DOI:** 10.3390/nu11102509

**Published:** 2019-10-18

**Authors:** James R. Myers, Lyle T. Wallace, Samira Mafi Moghaddam, Adrienne E. Kleintop, Dimas Echeverria, Henry J. Thompson, Mark A. Brick, Rian Lee, Phillip E. McClean

**Affiliations:** 1 Department of Horticulture, Oregon State University, Corvallis, OR 97331, USA; 2 Department of Horticulture, University of Wisconsin at Madison, Madison, WI 53706, USA; lyle.wallace@usda.gov; 3 Plant Resilience Institute, Department of Plant Biology, Michigan State University, East Lansing, MI 48824, USA; smafi@msu.edu; 4 Department of Plant Science, Delaware Valley University, Doylestown, PA 18901, USA; Adrienne.Kleintop@delval.edu; 5 RNA Therapeutics Institute, University of Massachusetts Medical School, Worcester, MA 01605, USA; dimas.echeverria@umassmed.edu; 6 Department of Horticulture and Landscape Architecture, Colorado State University, Fort Collins, CO 80523, USA; Henry.Thompson@colostate.edu; 7 Department of Soil and Crop Sciences, Colorado State University, Fort Collins, CO 80523, USA; Mark.Brick@colostate.edu; 8 Department of Plant Science, North Dakota State University, Fargo, ND 58105, USAphillip.mcclean@ndsu.edu (P.E.M.)

**Keywords:** common bean, *Phaseolus vulgaris*, flavonoids, association mapping, total phenolic compounds, genome wide association mapping

## Abstract

Snap beans are a significant source of micronutrients in the human diet. Among the micronutrients present in snap beans are phenolic compounds with known beneficial effects on human health, potentially via their metabolism by the gut-associated microbiome. The genetic pathways leading to the production of phenolics in snap bean pods remain uncertain. In this study, we quantified the level of total phenolic content (TPC) in the Bean Coordinated Agriculture Program (CAP) snap bean diversity panel of 149 accessions. The panel was characterized spectrophotometrically for phenolic content with a Folin–Ciocalteu colorimetric assay. Flower, seed and pod color were also quantified, as red, purple, yellow and brown colors are associated with anthocyanins and flavonols in common bean. Genotyping was performed through an Illumina Infinium Genechip BARCBEAN6K_3 single nucleotide polymorphism (SNP) array. Genome-Wide Association Studies (GWAS) analysis identified 11 quantitative trait nucleotides (QTN) associated with TPC. An SNP was identified for TPC on Pv07 located near the *P* gene, which is a major switch in the flavonoid biosynthetic pathway. Candidate genes were identified for seven of the 11 TPC QTN. Five regulatory genes were identified and represent novel sources of variation for exploitation in developing snap beans with higher phenolic levels for greater health benefits to the consumer.

## 1. Introduction

The need for transdisciplinary efforts to bridge the gap between production agriculture and public health nutrition is clear [1]. A particularly useful channel through which this can occur is among scientists working in horticulture and in human health since horticulture is a science familiar to most consumers and is practiced by many. In particular, it is critical for nutritionists to recognize the many factors that are involved in providing consumers with health-beneficial cultivated varieties (cultivars) of a crop and that potential value-added health benefits may result if cultivar-specific food labeling becomes a reality [2,3]. From a horticultural perspective, this creates the opportunity for niche markets and for the direct marketing of specific cultivars to the consumer [4,5]. One crop that has received limited attention relative to its health benefits, particularly those related to phenolic compounds, is snap bean (*Phaseolus vulgaris* L.), which is the topic of this investigation.

Snap beans (also known as garden, green, string, or French beans) are a form of common bean. As opposed to dry bean, in which the mature seeds are eaten, the immature pods and seeds of snap beans are consumed as a vegetable. Dry beans are considered a good source of protein, complex carbohydrates, soluble fiber, folic acid and vitamin B. In contrast, because of their higher water content (~90% water compared to ~12%), snap beans have lower protein and total carbohydrate levels, but they possess certain vitamins and carotenoids that dry beans do not have or have only in trace amounts. These include vitamin C, β-carotene, lutein and zeaxanthin, α-tocopherol, and phylloquinone [6]. About 1.5 million Mg of snap beans are produced annually on a global basis [7]. The US accounts for 71% of world production of snap beans, and Americans consume about 3.0 kg per person per year [8], and as such, snap beans are a significant source of vitamins, carotenoids and other secondary plant compounds in the human diet. 

Among secondary plant compounds are phenolics and related flavonoids. Phenolics constitute a large class of phytochemicals with over 8000 individual compounds having been identified. Structurally, they are characterized by an aromatic ring with one or more hydroxyl groups [9,10,11]. Phenolics and flavonoids are further divided into flavonols, flavones, flavan-3-ols, anthocyanidins, flavanones, isoflavones, and other minor subclasses, and nonflavonoids, which include phenolic acids, hydroxycinnamates, and stilbenes [9]. 

In general, phenolics are associated with various human health benefits including acting as anticarcinogens, antimicrobial agents, anti-inflammatories and improving vascular health, possibly by their metabolism via the gut-associated microbiome [12]. Total phenolic content has a strong antioxidant activity in legumes [13,14,15]. Dry bean consumption is associated with a reduced risk of diabetes, heart disease, colon cancer, and prostate cancer, and the mitigation of obesity [16,17,18,19]. These benefits may accrue in part from the phenolic content of seeds. We know less about the health benefits of snap beans, and to our knowledge, no human health studies similar to those conducted for dry beans have been reported. 

Several research groups have characterized snap beans for various phenolics. Abu-Reidah et al. [20] found more than 72 compounds including 10 phenolic acids and 59 flavonoids. Predominant among these were the flavonol glucosides quercetin and kaempferol along with the phenolic acids (+) catechin and (−) epicatechin, chlorogenic and protocatechuic acids, as well as procyanidins [21], [22]. Flavonoid levels in processed fresh snap beans ranged from 20 to 51 mg kg^−1^ [23]. In a study of six wax and green bean cultivars, flavonols levels ranged from 19 to 184 µg g^−1^ fresh weight (FW) for quercetin derivatives and 6 to 15 µg g^−1^ for kaempferol derivatives [24]. Kleintop et al. [25] examined the total phenolic content (TPC) in fresh and frozen pods of a snap bean diversity panel of 149 accessions. They found more than a four-fold difference among cultivars, which ranged from 0.29 to 1.31 mg g^−1^ gallic acid equivalents (GAE). Higher levels were generally associated with cultivars that had colored seeds and a purple or pink flower color. 

Snap beans grown for processing vs. the fresh market have different quality characteristics that may affect TPC. Processors require that cultivars be white-seeded to ensure that no water-soluble flavonoids are produced in any plant parts including the pods as they will leach into the liquor in canned beans and present as a colored ring in the testa in cut frozen beans, thereby reducing quality [26]. The standards for fresh market beans are less stringent, and while colored-seeded cultivars are acceptable, they are generally brown-seeded (produced by flavonols) rather than black or purple (a result of anthocyanins) because the former are less noticeable in the pod. Brown-seeded cultivars will generally have pods that are green in appearance, while purple- or black-seeded cultivars may have solid purple or striped purple and green pods. 

In common bean, several gene combinations may give a white seed color, but in the commercial trade, essentially all white-seeded snap beans possess the recessive *p* gene. This gene is a major switch in the flavonoid biosynthetic pathway, with the dominant form allowing the expression of other color genes while the recessive allele blocks expression. *P* is located on Pv07 (Table 1) and appears to be a basic helix loop helix (bHLH) MYB transcription factor [27]. Other effects of *p* are a reduced lignin content in seeds and greater sensitivity to imbibitional injury and reduced germination and emergence if the seed is not protected with fungicides [28,29]. The types of flavonols and anthocyanins produced and their expression are controlled by more than 10 additional genes [30]. These can be divided into those that act in the flavonoid biosynthetic pathway and those that affect spatial and temporal expression [31]. Most but not all of these factors have been mapped with varying degrees of accuracy [27,32,33] (Table 1). Little is known about the genetic control of phenolic acids in common bean. Kleintop et al. [25] observed a substantial difference in TPC in colored-seeded vs. white-seeded accessions in a snap bean diversity panel, but within white-seeded types, variation in TPC was also noted, suggesting that there is a genetic variation for colorless phenolic compounds. 

Genome-Wide Association Studies (GWAS) are a means of identifying and mapping quantitative trait loci (QTL) associated with phenotypic traits in plant populations. GWAS relies on linkage disequilibrium in individuals in regions in which QTL reside. A number of association-mapping studies have been conducted on common bean. These have analyzed traits associated with phenological variation [36,37,38], agronomic performance [39,40], biological nitrogen fixation [41], plant architecture [42], seed quality [43,44], and abiotic [45,46] and biotic [47,48,49], stress variation. None have focused on traits specific to snap bean. Association studies of phenolics in non-legume crops have focused primarily on cereals such as barley [50,51], whole rice [52,53,54], dehulled rice [55], wheat [56], sorghum [57], and maize [58], with single studies of tomato [59], rapeseed [60], and olive [61]. 

Considering the fairly substantial intake of snap beans in the United States, improving the phenolic content of snap beans could have a meaningful impact on human health. Therefore, the objective of this study was to conduct GWAS on a snap bean diversity panel that had been evaluated for total phenolic content. We wanted to 1) identify genomic regions associated with variation in TPC, 2) determine whether known color genes co-segregate with these regions, and 3) identify potentially novel genes that regulate TPC, especially in white-seeded cultivars, as a basis for further improving snap bean phenolic content.

## 2. Materials and Methods

### 2.1. Snap Bean Diversity Panel

One-hundred and forty-nine snap bean cultivars comprising the Bean CAP Snap Bean Diversity Panel were grown at Oregon State University (OSU) at Corvallis, OR during the 2010 growing season. The cultivars were planted into three field replicates using a randomized complete block design. Seeds were planted in 76 cm rows with a within row spacing averaging 5 cm in plots of 4 m in length. For fertility, 168 kg·ha^−1^ of 10N-4.4P-8.3K was banded in a row at planting. Weeds were controlled with application of 0.9 kg·ha^−1^ of Dual (Syngenta, Basel, Switzerland) S-metolachlor pre-emergent herbicide supplemented by mechanical cultivation. Western twelve-spotted cucumber beetles (*Diabrotica undecimpunctata undecimpunctata*) were controlled in young seedlings by the application of 1.0 L·ha^−1^ of Sevin (Bayer, Research Triangle Park, NC) 1-naphthyl N-methylcarbamate insecticide. Plots received approximately 2.5 cm of water per week from planting to harvest via solid set overhead sprinklers. Fresh pods of the predominant sieve size [25] for each cultivar were handpicked in the field at OSU, brought to the field laboratory, evaluated for fresh pod color and frozen at −20 °C for TPC analysis. Harvest maturity was determined by observing seed development and initiating harvest when seeds in the most mature pods had reached a length of approximately 10 mm. Approximately 10 pods per plot (50–100 g) were randomly selected from the plants within a plot. Frozen samples were shipped on dry ice to Colorado State University where they were stored at −80 °C until TPC analysis.

### 2.2. Total Phenolics Analysis

The TPC was analyzed spectrophotometrically using the Folin–Ciocalteu colorimetric method [62] adapted to a microplate format. The assay is based on the color reaction of phenolic compounds with Folin–Ciocalteu phenol reagent that absorbs light at 765 nm. The TPC is a measure of the reducing capacity of a sample through electron transfer reactions, expressed as gallic acid equivalents (GAE). All TPC values are expressed as milligrams GAE per gram of FW of tissue. Pod sample extracts were prepared according to the methods by Xu and Chang [14], and details of the procedure can be found in [25]. 

### 2.3. Phenotyping

Phenotypic traits used in the GWAS included flower and fresh pod color. Flowers were classified as white (*vv*) pink (*v^lae^v^lae^*) and purple (*VV*) and were coded to a three-point scale where 1 = white, 2 = pink and 3 = purple. The external color of fresh pods was quantified using a colorimeter (Minolta BC-10; Konica Minolta Sensing Americas, Ramsey, NJ) with data reported using the CIE (Commission Internationale de l’Eclairage) L*, a*, and b* scale [63]. L* is a measure of the brightness and ranges from 0 (black) to 100 (white). The a* vector represents colors from green (−) to red (+), and the b* vector measures blue (−) to yellow (+) in relative numbers [63].

### 2.4. Genotyping

A modified cetyl trimethyl ammonium bromide (CTAB) procedure was used to extract genomic DNA, and the resulting DNA samples were analyzed on an Illumina Infinium Genechip as described by [64]. The single nucleotide polymorphism (SNP) array utilized was composed of 10,546 allele-specific probes. The raw data was initially processed on GenomeStudio (v2.0.4) software (Illumina, San Diego, CA, USA). Two marker SNP positions contained greater than 20% missing data and were removed from the study. All missing data for the remaining SNPs was imputed using fastPHASE software (v1.4), including heterozygous SNPs which were treated as missing data. SNPs not assigned to a genomic position in Phytozome 12 (*Phaseolus vulgaris*, version 2.1) were removed from the study, resulting in 10,073 remaining SNPs.

### 2.5. Genome-Wide Association Mapping

GWAS was performed in R statistical software, version 3.3.2, (https://www.r-project.org/) using FarmCPU, version 1.02 (http://www.zzlab.net/FarmCPU/) and GAPIT, version 3.0, (https://www.maizegenetics.net/gapit) R packages, with data formatted in Microsoft Excel 2016 (https://office.microsoft.com/excel/) or TextPad version 7.5.1 (www.textpad.com). The principal component analysis utilized in the GWAS was generated with Tassel, version 5.2.24, (https://www.maizegenetics.net/tassel).

The iterative fixed and random model of FarmCPU was chosen as the primary model for GWAS with an added covariate of 1 PC. Analysis was performed in R using the FarmCPU source code provided by [65]. Data sets were also analyzed using an Efficient Mixed Model Association (EMMA) in GAPIT to only control for individual relatedness. For both models, a minor allele frequency (MAF) of 0.05 was used, which reduced the SNP number to 8008. The negative log *P*-value cutoff on the Manhattan plots was 4.912, representing a Bonferroni cutoff threshold using α = 0.05 to denote SNPs as significant. For the FarmCPU analysis, an additional line of code not shown in the manual was used to generate a complete list of SNPs in the results document: threshold. output = 1. 

### 2.6. Candidate Gene Search

Literature on the known color genes of common bean were compiled to identify which portion of the phenolic biosynthetic pathway the gene might be involved in, and to identify known markers and their map positions (Table 1). For the sequence tagged site (STS) markers of McClean et al. [33], forward and reverse primer sequence matches were identified in Phytozome12 (*Phaseolus vulgaris*, version 2.1), and the chromosome and physical position obtained. A key word search for regulatory [66,67,68,69,70] and structural [35,71] genes in the phenolic biosynthetic pathway was conducted in supplemental tables obtained from [72] (updated to *P. vulgaris* genome sequence ver. 2.1) containing a list of *Phaseolus vulgaris* gene identifiers and their *Arabidopsis thaliana* gene homologues, gene symbols and annotations. The physical position of these candidate genes was compared to the physical position of SNPs identified by GWAS. A distance of approximately 350 kb upstream and downstream of the associated SNPs was used when considering the proximity of identified genes. This distance was established based on 1) the observed distance between SNP markers associated with the white- vs. colored-seed phenotype and the location of the candidate gene for *p* [27], and 2) the slow decay of linkage disequilibrium that is characteristic of highly self-pollinated species [73], and specifically common bean [74,75,76].

## 3. Results

### 3.1. Phenotypic Data

Detailed analysis of the phenotypic data can be found in Kleintop et al. [25] for TPC, pod color and flower color. TPC ranged from 0.29 to 1.31 mg g^−1^ GAE, with the highest levels being associated with colored flowers and pigmented pods. However, a two-fold variation (0.29–0.64 mg·g^−1^ GAE) was observed in TPC among white-flowered and -seeded accessions, implying variation in colorless phenolic compounds. The proportion of individuals with colored flowers and/or seeds is 16%, leading to relatively few individuals having a major influence on phenolics profiles. Pod color is also affected by non-phenolic compounds that affect the degree of greenness. Accessions with the lightest colored pods possessed the wax pod trait conditioned by *y* or were Refugee or Romano types [77].

### 3.2. Genome-Wide Association Study

A total of 30 quantitative trait nucleotides (QTN) significantly associated with all traits using a Bonferroni cut-off of 4.9 (using α = 0.05) were identified by both GAPIT and FarmCPU analyses (Table 2, Table 3 and Table 4). QTN were found on all chromosomes except Pv06 and Pv08. Two-thirds of the QTN were identified by FarmCPU and the remainder by GAPIT. QTN could be placed into about 21 groups with intervals of up to ~290 kb. Eleven SNPs were associated with TPC, six with pod color and 13 with flower color. The same SNP was associated with b* (B*3.2) and TPC (TPC3.1) on Pv03, with an effect of a negative shift (−8.05) in the yellow–blue axis being associated with increasing (0.27 mg·g^−1^ GAE) TPC (Table 2 and Table 3). TPC and flower color had two co-localized peaks found on Pv09 (Table 2 and Table 4). Both FarmCPU and GAPIT identified the same SNP on Pv07 for *P*, but the SNP identified by GAPIT did not meet the Bonferroni cutoff. 

### 3.3. Total Phenolic Content QTN

**Figure 1:**
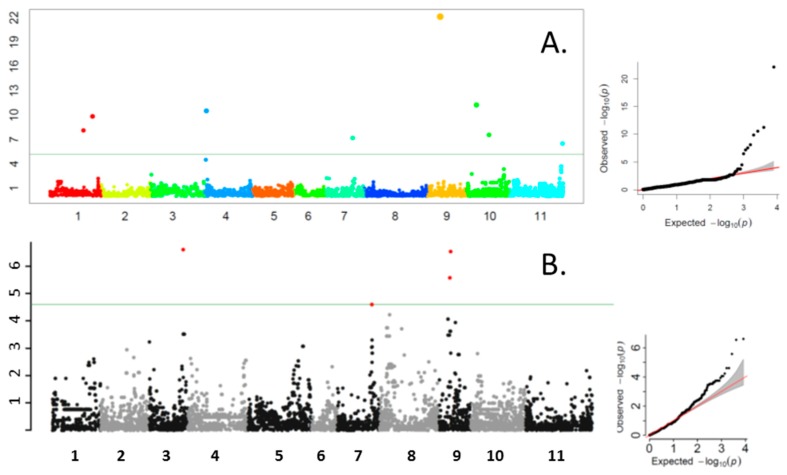
Manhattan and quantile-quantile (QQ) probability plots for total phenolic content of pods from the Bean CAP snap bean diversity panel showing the location of SNPs significantly associated with total phenolic content quantitative trait nucleotides. X axis: chromosome number; Y axis: *-log_10_ (P)*. Output from FarmCPU (**A**) and GAPIT (**B**), with a horizontal line representing the Bonferroni cut-off (α = 0.05).

Eleven SNPs were associated with QTN for TPC (Table 2, Figure 1). Two separate QTN were observed on each of Pv01 and Pv10, and Pv09 had three separate QTN; the remainder had one QTN per chromosome. TPC7.1 appears to be associated with the *P* locus, which has been located to a region from 28,752,132–28,774,743 for the gene models Phvul.007G171333 and Phvul.007G171466 (Table 1). The SNP identified by FarmCPU was located about 320 kb from the candidate gene. A second SNP (ss715647650, P = 2.51 × 10^−05^) identified by GAPIT was located about 6 kb from the SNP identified by FarmCPU but did not meet the statistical threshold for significance. Two other color genes were located in the same general regions as significant QTN. TPC 9.1 and *T* were in proximity on Pv09 as were TPC10.1 and *Ana*. However, the distances between TPC SNPs and gene markers were about 427 and 433 kb, respectively, and were outside of our designated window for linkage disequilibrium decay. No other color genes for which the physical position could be estimated appear to be associated with TPC QTN. With the exception of TPC7.1 (MAF = 0.29), most QTNs had low minor allele frequencies ranging from 0.05–0.09 (Table 2). 

### 3.4. Pod Color QTN

Pod color SNPs were associated with all three parameters of the L*a*b* scale (Table 3, Figures S1–S4). One SNP was associated with a* (red–green axis), two with L* (light–dark), and three with b* (blue–yellow axis) on three chromosomes. Three significant QTN identified by GAPIT on Pv02 form a 224kb interval spanning a region from 542,087–766,293 bp that includes SNPs for both L* and a*. The same SNP for A*2.1 was also associated with L*, but did not meet the Bonferroni cutoff. We have previously mapped the wax pod (*y*) locus to Pv02, which is 23 kb from the A*2.1 QTN. A possible candidate gene for this trait is Phvul.002G004400, located at 516,568–518,904kb [77]. It has been annotated in Arabidopsis as a pentatricopeptide repeat-containing protein, which is targeted to organelles and has been shown to cause cytoplasmic male sterility when it interacts with mitochondria and albinism when targeted to chloroplasts [78]. Our conclusion is that this pod color trait does not have any relationship to the genetic control of the phenolics biosynthetic pathway.

Some of the other pod color SNPs appear not to be associated with phenolics. For example, neither B*3.1 or B*5.1 show a relationship to TPC. However, as mentioned above, the SNP underlying B*3.2 is identical to TPC3.1, suggesting that this QTN is associated with a shift in pod color. None of the regions associated with pod color appeared to be associated with regions where classic bean color genes have mapped. 

### 3.5. Flower Color SNPs

Thirteen SNPs on three chromosomes were associated with flower color (Table 4, Figures S5 and S6). Most were identified by FarmCPU, with three found by GAPIT. Two QTN were located on Pv01, four on Pv03 and three on Pv09. The first three groups on Pv03 spanned 12.9, 21.2 and 88.1 kb, respectively. FC3.1 was located about 255 kb from TPC3.1 and B*3.2. There were no significant QTN on Pv06 or Pv07 where genes are known to reside that qualitatively control flower color. 

### 3.6. Candidate Genes in Proximity to Traits

A total of 545 gene models were found within 350 kb of the 11 TPC QTN (Table 5). Four-hundred and sixty-four of these had annotations for Arabidopsis homologues, while the function of 81 was unknown. Among all gene models, 19 appeared to be related to the phenolics biosynthetic pathway-based annotation, mainly as regulatory genes. The number of gene models within most intervals ranged from 52 to 67, but intervals on Pv01 and Pv10 were more sparsely populated, with 13–19 gene models observed. The greatest number of potentially phenolic-related gene models was found on Pv04, resulting from a cluster of 10 Cytochrome P450 genes arranged in tandem (Table 6). 

The potential phenolics pathway gene models included bHLH DNA-binding superfamily proteins, cytochrome P450 (various families and subfamilies), myb-like transcription factor family proteins, NAD(P)-binding Rossmann-fold superfamily protein, and Transducin/WD40 repeat-like superfamily protein (Table 6). 

No candidate genes were found in proximity to TPC1.2, TPC3.1, TPC10.1 and TPC10.2 (Table 6). For TPC1.1, a single candidate gene was identified as Phvul.001G121200, located 83,056 bp distant from the SNP. The Arabidopsis homologue for Phvul.001G121200 is AT2G41130.1 which encodes the bHLH factor BHLH106. In Arabidopsis, it is also designated *STC8* (*salt tolerant callus 8*) which has been shown to regulate a hyperosmotic salinity response in Arabidopsis [79], but in *Vitus vinifera*, BHLH106 is associated with the light-induced negative regulation of the anthocyanin pathway [80]. 

For TPC4.1, a cluster of 10 cytochrome P450, family 82, subfamily C, polypeptide 4 gene models (Phvul.004G021100 - Phvul.004G0220000 were found located from 269 to ~338 kb from the SNP. The corresponding Arabidopsis homologue is AT4G31940.1 (CYP82C4) (Table 6). In Arabidopsis, this gene is involved in iron uptake and transport, but in basil (*Ocimum basilicum*), it catalyzes the 7-*O*-methylation of flavones [81]. 

TPC7.1 is represented by two gene models that reside about 6.5 kb from each other (Table 6). TPC7.1 is associated with Phvul.007G171333 and Phvul.007G171466, which appear to be part of the same gene model and are ~318–345 kb from the SNP. These gene models have been shown to be homologous to the Arabidopsis gene model AT4G09820.1 which encodes for the basic helix loop helix DNA binding super family protein BHLH42 [27]. The Arabidopsis gene is *transparent testa 8* (*TT8*), and in *P. vulgaris*, this controls flavonoid expression via the *p* locus. 

For TPC9.1, two *P. vulgaris* genes of similar function are located within 28–30 kb of the SNP (Table 6). These are Phvul.009G069401 and Phvul.009G069500 which correspond to AT5G19440.1 and AT1G51410.1, respectively, which are uncharacterized NAD(P)-binding Rossmann-fold superfamily proteins. Among other functions, these types of proteins have been found to be involved in the flavonoid pathway [82].

TPC9.2 and TPC9.3 are located at 242,880 and 32,540, respectively, from the same candidate gene (Phvul.009G077300), which corresponds to the Arabidopsis homologue AT5G56840.1 (Table 6). This gene encodes an myb-like transcription factor family protein but remains uncharacterized in Arabidopsis. However, in tea (*Camellia sinensis*), an myb homologous to AT5G56840.1 is involved with epigallocatechin-3-gallate biosynthesis [83]. 

Two candidate genes of similar function were found for TPC11.1. These were Phvul.011G212600 and Phvul.011G212800, located within about 3 kb of each other and homologous to the same Arabidopsis gene model AT2G45400.1 (Table 6). The Arabidopsis gene has been named *BEN1* (*bri1-5 enhanced 1*) and has been characterized as an NAD(P)-binding Rossmann-fold superfamily protein that encodes a dihydroflavonol 4-reductase (DFR)-like protein [82]. In Arabidopsis, *BEN1* has been shown to be involved in the brassinosteroid metabolic pathway; however, *BEN1* is a member of a small gene family that also includes dihydroflavonol 4-reductase and anthocyanidin reductase (*BAN*). In *Brassica rapa*, the homologous gene model (Bra004907.1) is involved in the flavonoid biosynthetic pathway [84]. 

## 4. Discussion

Because anthocyanins are well known for their red-to-blue color, and flavonols are associated with yellow-to-brown pigments, we classified snap bean accessions for flower and seed color as well as obtaining a color profile for pods, in addition to measuring TPC. However, other phenolic compounds may be colorless and may account for the two-fold variation in TPC that was observed apart from that attributed to flavonoids. Kleintop et al. [25] found moderate significant correlations between TPC and a* (0.40) and between TPC and b* (−0.34). There was essentially no correlation between TPC and L*. These results indicate that, of the parameters evaluated here, a* and b* are more likely to be associated with QTN for TPC than L*. 

In this association study, we found 11 QTN for TPC located on seven chromosomes of common bean, but only one QTN (TPC7.1) was associated with a known major gene that has a large phenotypic effect. In addition, the QTN was located 324–245kb from the gene models associated with this trait. In a dry x snap bean mapping population, Hagerty et al. [32] found SNP markers associated with *p* that were located ~17.2Mb from the candidate genes identified by [27]. While the slow rate of LD decay in common bean may influence marker-gene associations, *p* is not located in a low recombination region of Pv07, and so there may be other explanations for why distances between this locus and markers are rather large. 

Compared to the other phenotypic categories, there were relatively few pod color SNPs, and most were not likely to be associated with pigmented flavonoids. Based on the moderate correlations between pod color and total TPC identified in [25], we would expect that a* and b* would be most likely to be associated with phenolics; in this case, however, a* (and L*) are associated with the wax bean trait, which is marked by a deficit of chlorophyll and not variation in TPC. Three b* QTN may be related to phenolic-based pod color, which is supported by B*3.2-associated SNP also underlying TPC 3.1. In addition, FC 3.1 was located within the LD decay window at 255 kb from B*3.2 and TPC 3.1. These results all support the idea that a QTN affecting flower and pod color and phenolic content resides in this region of Pv03. This QTN does not appear to be related to any known color genes. All b* QTN may represent novel genes affecting pod color that have not been identified in classical genetic studies.

The findings for flower color QTN were unexpected. We predicted that we would find two or three QTN associated with *P* and *V*, and possibly *T*, because these genes are known to modify flower color. As described above, *P* is located on Pv07, whereas *V* has previously been placed on Pv06 (Table 1). Dominant *P* is required for observing segregation at *V* and *T*, which means that the number of genotypes that could be classified for these genes was small, and any associations that might have been found in GWAS would be discarded because they fell below a 5% MAF cut-off. The 13 QTN associated with flower color appeared to be novel and it is difficult to see how these were identified during GWAS, since the classification of genotypes was based on a three-point scale. This may point to the need for the better quantification of flower color. The pink class in particular can sometimes be difficult to classify as it varies from very similar to white on one hand to purple on the other hand. One QTN (FC 9.1) had the same underlying SNP as a TPC QTN (TPC 9.1), which suggests that the pigment difference in that case was real. 

The data used in this study represent a single environment. In our previous study [25], we examined TPC over two years in a subset of pole and bush bean accessions. We found no genotype by the year of interaction for the indeterminate pole bean group, and a weak genotype by the year of interaction for determinant bush accessions. TPC levels were significantly higher in the second year, and the interaction term for the bush bean group was driven by changes in magnitude—particularly by one accession—rather than by cross-over interaction. Based on these results, we concluded that the genotype by environment effect on TPC in this population was relatively small, and that we could proceed with a GWAS analysis based on the single environment. In some cases, the magnitude of the TPC’s effects from GWAS in the present situation was small and would need to be verified in more environments to provide validation. With additional resources, it would be useful to conduct a more extensive analysis of environmental effects as well as other factors, such as pod maturity, on TPC.

While most structural genes in the phenolic biosynthetic pathway have been identified, the regulatory control of the pathway is in the early stages of analysis. Some regulatory factors have been identified, with the majority of these characterized in Arabidopsis [66,67,68,69,70,71] and grape [80]. One difficulty is that a function in one species is not necessarily the same in other species, especially where particular biosynthetic pathways may be missing (such as the glucosinolate biosynthetic pathway found in Brassicaceae but not in most other plant families). This works both ways, in that regulatory genes identified as being in the phenolics biosynthetic pathway in Arabidopsis may be involved in different pathways in another organism and vice versa. The lack of functional studies for regulatory genes in legumes in general and common bean in particular hampers the association of putative phenolics biosynthetic pathway regulatory genes with gene function. It may be that for several QTN (TPC 1.2, TPC 3.1, TPC 10.1 and TPC 10.2), novel genes may be involved. In the present case, these may take the form of unrecognized regulatory or structural genes, or genes that have yet to receive annotation in either model systems, such as Arabidopsis, or in the species of interest. The same may be true for those QTN for which we did identify potential genes. There may be another unrecognized candidate gene in the vicinity of the QTN. The ultimate proof will be research that demonstrates gene function. 

The identification of QTN for health-promoting phenolics will be an asset to plant breeders. High-throughput molecular markers can readily be derived from SNP positions that have been identified as associated to a trait. Marker-assisted selection, along with simple colorimetric assays as described by [25], will result in a powerful set of tools for selecting for these traits. This will not only benefit fresh market beans but also processing beans because the white-seeded varieties required by processors can be bred for higher TPC. As previous research [25] has shown, the cultivar McCaslan No. 42 may be highly valuable with regard to developing white-seeded processing beans with increased TPC levels, and molecular markers will greatly assist in moving these traits into other snap bean backgrounds. Considering the fairly substantial intake of snap beans in the United States, improving the phenolic content of snap beans could have a meaningful impact on human health.

## Figures and Tables

**Table 1:** Location in the *Phaseolus vulgaris* physical map of linked markers and candidate genes for color and patterning genes associated with the flavonoid biosynthetic pathway compiled from existing literature and databases ^1,6^.

Gene ^2^	Chrom. ^3^	Marker/Gene Model	Linkage ^4^	Start	End	Length ^5^
			cM	bp		
*B*	2	ss715645998	0.0	48,634,623	48,634,743	121
*Z*	3	OAM10560	1.4	31,467,887	31,467,909	22
*G*	4	OAP7850	0.0	30,171,480	30,172,322	842
*G*	4	OAP31400	0.0	30,172,301	30,172,322	21
*G*	4	OU14900	0.0	31,093,347	31,093,369	22
*V*	6	OD12800	0.0	9,288,398	9,288,419	21
*P*	7	Phvul.007G171333 + Phvul.007G171466	−	28,752,132	28,774,743	22,611
*Gy*	8	OW17600	1.6	3,230,983	3,231,005	22
*C*	8	OAP2700	4.9	9,694,328	9,695,007	679
*T*	9	OM19400	1.4	11,731,567	11,731,944	377
*Ana*	10	OM9200	5.4	11,563,621	11,563,800	179
*L*	10	OL4525	1.2	41,443,673	41,443,694	21

^1^ Original linkage map and primer sequence data from [27,32,33]. ^2^ Color and patterning genes of common bean. *Ana*: modifies expression of *T*, *B*: greenish-brown seed testa, *C*: complex locus controlling seed testa patterning, *G*: yellow-brown seed testa, *Gy*: greenish-yellow seed testa, *L*: inhibitor of expression of partial coloring of *t*, *P*: recessive form conditions white seed testa and flowers, *T*: recessive form conditions partially colored testa, *V*: conditions violet seed testa, *Z*: modifier of expression of *T*. See [30] for additional detail. ^3^ Chromosome. ^4^ Linkage distance between the color/patterning gene (determined phenotypically) and marker as determined in [32,33]. ^5^ Length in base pairs refers to the length of the candidate gene (as in the case of *p*) or the length of a marker sequence linked to a color/patterning gene. ^6^ Not shown is *Rk* (recessive red seed testa), whose location is controversial; it has been located to both Pv01 [34] and Pv02 [35].

**Table 2:** Chromosome and physical location of single nucleotide polymorphisms significantly associated with total phenolic content (TPC) in pods of the Bean CAP snap bean diversity panel.

QTN Designation ^1^	Method	ss ID No. ^2^	Chrom. ^3^	Position (bp)	Prob. Value	*-Log_10_ P*	MAF ^4^	SNP ^5^
TPC1.1	FarmCPU	715648077	1	33,470,635	7.952E-09	8.10	0.05	C-T
TPC1.2	FarmCPU	715646871	1	42,698,218	1.357E-10	9.87	0.07	T-C
TPC3.1	GAPIT	715646623	3	40,370,083	2.448E-07	6.61	0.05	T-C
TPC4.1	FarmCPU	715647815	4	2,190,413	2.912E-11	10.54	0.07	G-A
TPC7.1	FarmCPU	715647649	7	28,427,257	6.783E-08	7.17	0.29	T-C
TPC9.1	GAPIT	715647263	9	12,159,056	2.614E-06	5.58	0.09	C-T
TPC9.2	GAPIT	715646560	9	12,776,010	2.872E-07	6.54	0.08	A-G
TPC9.3	FarmCPU	715646559	9	13,053,019	7.182E-23	22.14	0.08	G-A
TPC10.1	FarmCPU	715639693	10	11,130,696	5.660E-12	11.25	0.06	T-C
TPC10.2	FarmCPU	715643755	10	23,719,802	2.746E-08	7.56	0.06	C-T
TPC11.1	FarmCPU	715650328	11	52,968,750	3.222E-07	6.49	0.05	T-C

^1^ Quantitative trait nucleotide. ^2^ National Center for Biotechnology Information (NCBI) Assay SNP ID. ^3^ Chromosome. ^4^ Minor allele frequency. ^5^ Allelic substitution at single nucleotide polymorphism (SNP) locus.

**Table 3:** Chromosome and physical location of single nucleotide polymorphisms significantly associated with fresh pod color in the Bean CAP snap bean diversity panel.

QTN Designation ^1^	Method	ss ID No. ^2^	Chrom. ^3^	Position (bp)	Prob. Value	*-Log_10_ P*	MAF ^4^	SNP ^5^
A*2.1	GAPIT	715646673	2	542,087	2.684E-06	5.57	0.11	G-T
L*2.1	GAPIT	715639371	2	729,615	1.324E-06	5.88	0.18	C-A
L*2.1	GAPIT	715639372	2	766,293	1.324E-06	5.88	0.18	C-T
B*3.1	FarmCPU	715649460	3	3,463,964	2.601E-09	8.58	0.07	T-C
B*3.2	GAPIT	715646623	3	40,370,083	1.790E-07	6.75	0.05	T-C
B*5.1	FarmCPU	715645117	5	887,360	3.322E-06	5.48	0.14	A-G

^1^ L*, A* and B* are vectors for the CIE L*a*b* color scale; numbers following the color parameter indicate chromosome number and QTN number on that chromosome. ^2^ National Center for Biotechnology Information (NCBI) Assay SNP ID. ^3^ Chromosome. ^4^ Minor allele frequency. ^5^ Allelic substitution at SNP locus.

**Table 4:** Chromosome and physical location of single nucleotide polymorphisms significantly associated with flower color in the Bean CAP snap bean diversity panel.

QTN Designation ^1^	Method	ss ID No.^2^	Chrom. ^3^	Position (bp)	Prob. Value	*-Log_10_ P*	MAF ^4^	SNP ^5^
FC1.1	FarmCPU	715648402	1	8,955,838	3.088E-21	20.51	0.06	A-C
FC1.2	FarmCPU	715645902	1	48,430,158	3.215E-10	9.49	0.09	T-C
FC3.1	FarmCPU	715646620	3	40,621,263	1.087E-08	7.96	0.07	T-C
FC3.1	FarmCPU	715646619	3	40,634,185	1.087E-08	7.96	0.07	A-G
FC3.2	FarmCPU	715650182	3	40,901,620	1.087E-08	7.96	0.07	T-C
FC3.2	FarmCPU	715650183	3	40,922,819	1.087E-08	7.96	0.07	A-C
FC3.3	FarmCPU	715639426	3	41,081,362	1.087E-08	7.96	0.07	C-T
FC3.3	FarmCPU	715639423	3	41,169,500	1.087E-08	7.96	0.07	T-G
FC3.4	FarmCPU	715640920	3	43,174,196	1.087E-08	7.96	0.07	A-C
FC9.1	GAPIT	715647263	9	12,159,056	2.326E-07	6.63	0.09	C-T
FC9.2	GAPIT	715646560	9	12,776,010	7.518E-08	7.12	0.08	A-G
FC9.3	FarmCPU	715646559	9	13,053,019	8.124E-21	20.09	0.08	G-A
FC9.3	GAPIT	715648638	9	13,339,943	1.028E-06	5.99	0.19	G-A

^1^ Quantitative trait nucleotide. ^2^ National Center for Biotechnology Information (NCBI) Assay SNP ID. ^3^ Chromosome. ^4^ Minor allele frequency. ^5^ Allelic substitution at SNP locus.

**Table 5:** Number of gene models within ± 350kb of quantitative trait nucleotides associated with total phenolic content from an association mapping study conducted using the Bean CAP snap bean diversity panel.

QTN Designation	Gene Models ± 350 kb of QTN	Annotated Gene Models	Putative Phenolics Related Gene Models	Unknown Gene Models
	no.			
TPC1.1	19	18	1	1
TPC1.2	61	55	0	6
TPC3.1	67	61	0	6
TPC4.1	67	61	10	6
TPC7.1	56	48	2	8
TPC9.1	65	55	2	10
TPC9.2	61	45	1	16
TPC9.3	65	50	1	15
TPC10.1	13	13	0	0
TPC10.2	19	18	0	1
TPC11.1	52	40	2	12

**Table 6:** Possible candidate genes in the phenolics pathway in proximity to quantitative trait nucleotides (QTN) associated with total phenolic content identified by association mapping in the Bean CAP snap bean diversity panel. Shown is the SNP physical position, distance between the SNP and a candidate gene, the gene model names for *P. vulgaris* and *A. thaliana*, and gene designation and function.

QTN ^1^	SNP Position	Distance ^2^	*P. vulgaris* Gene Model	Start	End	*A. thaliana* Gene Model	Gene Abbreviation	Gene Function
	bp			bp				
TPC1.1	33,470,635	83,056	Phvul.001G121200	33,553,691	33,555,719	AT2G41130.1	BHLH106, STC8	Basic helix-loop-helix (bHLH) DNA-binding superfamily protein
TPC1.2	42,698,218	--	No candidates	--	--	--	--	--
TPC3.1	40,370,083	--	No candidates	--	--	--	--	--
TPC4.1	2,190,413	269,553	Phvul.004G021100	2,459,966	2,462,674	AT4G31940.1	CYP82C4	Cytochrome P450, family 82, subfamily C, polypeptide 4
		275,490	Phvul.004G021200	2,465,903	2,467,865	AT4G31940.1	CYP82C4	Cytochrome P450, family 82, subfamily C, polypeptide 4
		283,486	Phvul.004G021300	2,473,899	2,476,387	AT4G31940.1	CYP82C4	Cytochrome P450, family 82, subfamily C, polypeptide 4
		289,437	Phvul.004G021400	2,479,850	2,482,645	AT4G31940.1	CYP82C4	Cytochrome P450, family 82, subfamily C, polypeptide 4
		298,067	Phvul.004G021500	2,488,480	2,491,886	AT4G31940.1	CYP82C4	Cytochrome P450, family 82, subfamily C, polypeptide 4
		304,827	Phvul.004G021600	2,495,240	2,497,544	AT4G31940.1	CYP82C4	Cytochrome P450, family 82, subfamily C, polypeptide 4
		312,938	Phvul.004G021700	2,503,351	2,505,988	AT4G31970.1	CYP82C2	Cytochrome P450, family 82, subfamily C, polypeptide 2
		321,346	Phvul.004G021800	2,511,759	2,513,935	AT4G31970.1	CYP82C2	Cytochrome P450, family 82, subfamily C, polypeptide 2
		325,444	Phvul.004G021900	2,515,857	2,518,683	AT4G31940.1	CYP82C4	Cytochrome P450, family 82, subfamily C, polypeptide 4
		338,085	Phvul.004G022000	2,528,498	2,531,156	AT4G31940.1	CYP82C4	Cytochrome P450, family 82, subfamily C, polypeptide 4
TPC7.1	28,427,257	324,875	Phvul.007G171333	28,752,132	28,766,155	AT4G09820.1	BHLH42, TT8	Basic helix-loop-helix (bHLH) DNA-binding superfamily protein
		345,156	Phvul.007G171466	28,772,413	28,774,743	--	--	Basic helix-loop-helix (bHLH) DNA-binding superfamily protein
TPC7.1	28,433,796	318,336	Phvul.007G171333	28,752,132	28,766,155	AT4G09820.1	BHLH42, TT8	Basic helix-loop-helix (bHLH) DNA-binding superfamily protein
		338,617	Phvul.007G171466	28,772,413	28,774,743	--	--	Basic helix-loop-helix (bHLH) DNA-binding superfamily protein
TPC9.1	12,159,056	30,719	Phvul.009G069401	12,122,701	12,128,337	AT5G19440.1	--	NAD(P)-binding Rossmann-fold superfamily protein
		28,423	Phvul.009G069500	12,129,317	12,130,633	AT1G51410.1	--	NAD(P)-binding Rossmann-fold superfamily protein
TPC9.2	12,776,010	242,880	Phvul.009G077300	13,018,890	13,020,479	AT5G56840.1	--	myb-like transcription factor family protein
TPC9.3	13,053,019	32,540	Phvul.009G077300	13,018,890	13,020,479	AT5G56840.1	--	myb-like transcription factor family protein
TPC10.1	11,130,696	--	No candidates	--	--	--	--	--
TPC10.2	23,719,802	--	No candidates	--	--	--	--	--
TPC11.1	52,968,750	287,108	Phvul.011G212600	53,255,858	53,257,674	AT2G45400.1	BEN1	NAD(P)-binding Rossmann-fold superfamily protein
		291,448	Phvul.011G212800	53,260,198	53,262,370	AT2G45400.1	BEN1	NAD(P)-binding Rossmann-fold superfamily protein

^1^ Quantitative trait nucleotide with letters designating trait (total phenolic content) and numbers following indicate chromosome number and QTN number on that chromosome. ^2^ Distance calculated from SNP to nearest end of gene model.

## Supplementary Materials

The following are available online at https://www.mdpi.com/2072-6643/11/10/2509/s1, Figure S1. Manhattan and QQ plots for the CIE color parameter L* of pods from a Genome-Wide Association Mapping study of the Bean CAP snap bean diversity panel. Output from GAPIT using the EMMA model with one PCA. The horizontal line represents the Bonferroni cut-off (α = 0.05). Figure S2. Manhattan and QQ plots for CIE color parameter a* of pods from a Genome-Wide Association Mapping study of the Bean CAP snap bean diversity panel. Output from GAPIT using the EMMA model with one PCA. The horizontal line represents the Bonferroni cut-off (α = 0.05). Figure S3. Manhattan and QQ plots for b* of pods from a Genome-Wide Association Mapping study of the Bean CAP snap bean diversity panel. Output from GAPIT using the EMMA model with one PCA. The horizontal line represents the Bonferroni cut-off (α = 0.05). Figure S4. Manhattan and QQ plots for the CIE color parameter b* of pods from a Genome-Wide Association Mapping study of the Bean CAP snap bean diversity panel. Output from FarmCPU with one PCA. The horizontal line represents the Bonferroni cut-off (α = 0.05). Figure S5. Manhattan and QQ plots for flower color from a Genome-Wide Association study of the Bean CAP snap bean diversity panel. Output from FarmCPU with one PCA. The horizontal line represents the Bonferroni cut-off (α = 0.05). Figure S6. Manhattan and QQ plots for flower color from a Genome-Wide Association study of the Bean CAP snap bean diversity panel. Output from GAPIT using the EMMA model with one PCA. The horizontal line represents the Bonferroni cut-off (α = 0.05). The original SNP data used in this study has been deposited in the ScholarsArchive^@^ OSU and can be retrieved at: https://ir.library.oregonstate.edu/concern/datasets/m900p1589.
