# Supplementary material for: Improving the Health Benefits of Snap Bean: Genome-Wide Association Studies of Total Phenolic Content
Source: Nutrients. 2019 Oct 18;11(10):2509. doi: 10.3390/nu11102509 (PMC6835575; doi:10.3390/nu11102509)
Supplement: Supplementary file 1 [file nutrients-11-02509-s001.pdf]

Supplemental Figures for Myers et al., Improving the health benefits of snap bean: Genome wide association studies of total phenolic content

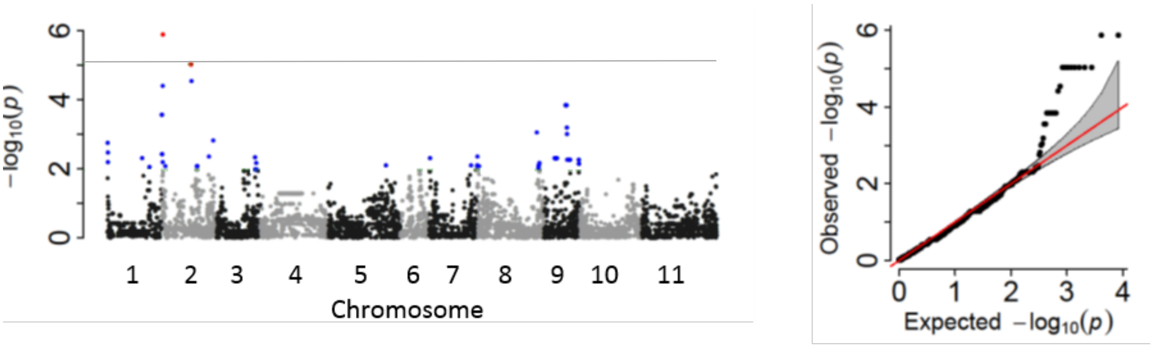

Figure S1. Manhattan and QQ plots for the CIE color parameter L* of pods from a genome wide association mapping study of the Bean CAP snap bean diversity panel. Output from GAPIT using EMMA model with one PCA. Horizontal line represents the Bonferroni cut-off ($\alpha = 0.05$).

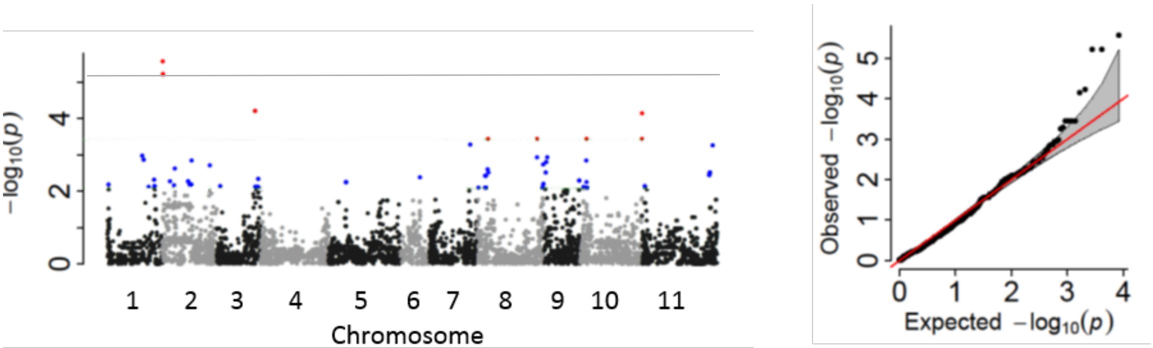

Figure S2. Manhattan and QQ plots for CIE color parameter a* of pods from a genome wide association mapping study of the Bean CAP snap bean diversity panel. Output from GAPIT using EMMA model with one PCA. Horizontal line represents the Bonferroni cut-off ($\alpha = 0.05$).

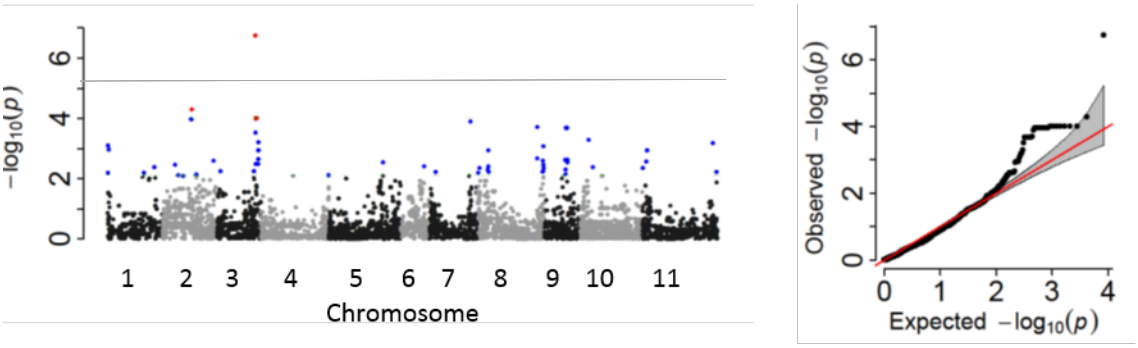

Figure S3. Manhattan and QQ plots for b* of pods from a genome wide association mapping study of the Bean CAP snap bean diversity panel. Output from GAPIT using EMMA model with one PCA. Horizontal line represents the Bonferroni cut-off ($\alpha = 0.05$).

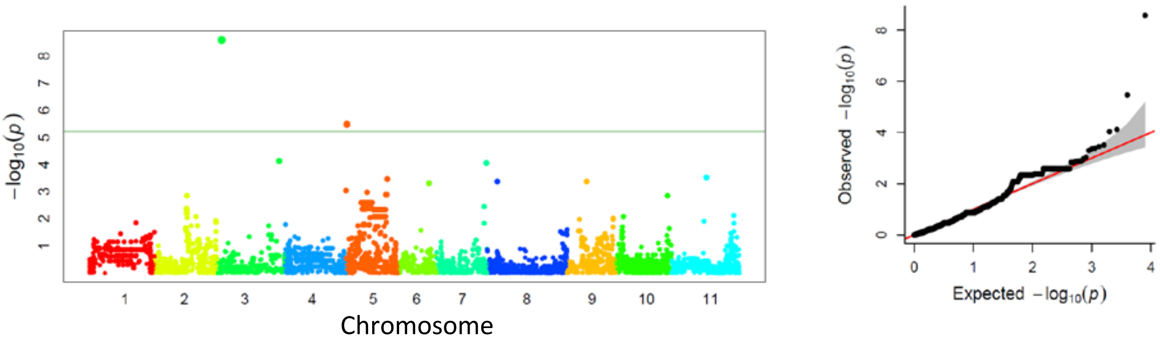

Figure S4. Manhattan and QQ plots for the CIE color parameter b* of pods from a genome wide association mapping study of the Bean CAP snap bean diversity panel. Output from FarmCPU with one PCA. Horizontal line represents the Bonferroni cut-off ($\alpha = 0.05$).

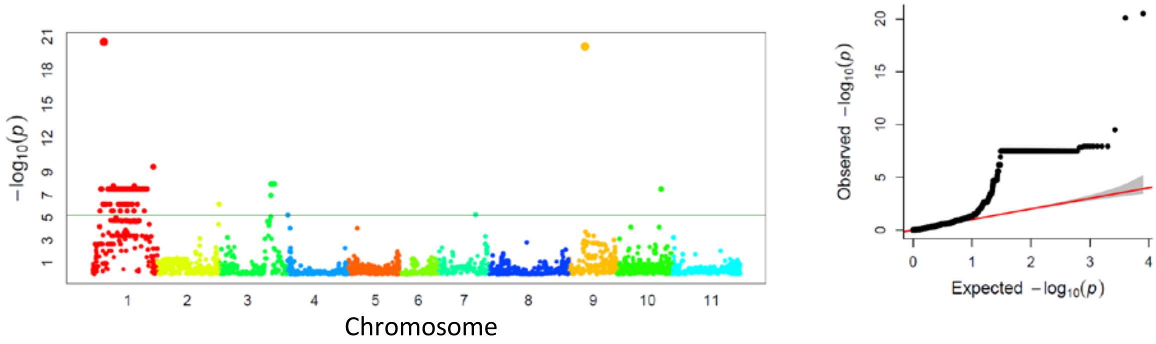

Figure S5. Manhattan and QQ plots for flower color from a genome wide association study of the Bean CAP snap bean diversity panel. Output from FarmCPU with one PCA. Horizontal line represents the Bonferroni cut-off ($\alpha = 0.05$).

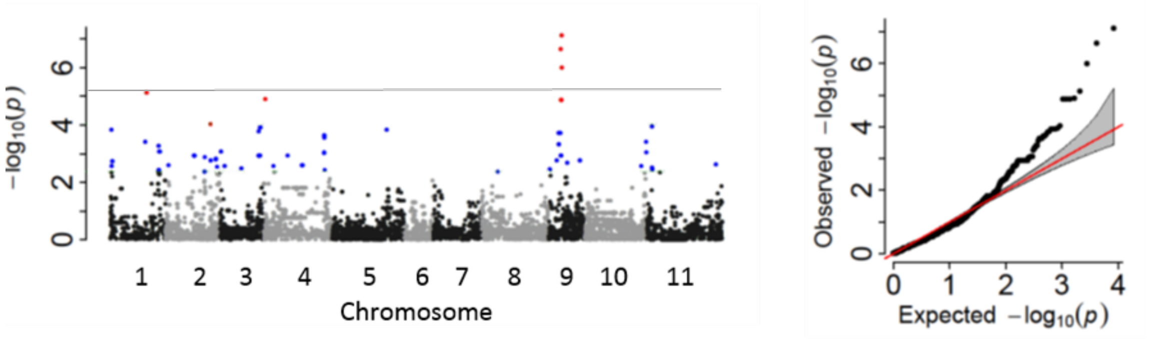

Figure S6. Manhattan and QQ plots for flower color from a genome wide association study of the Bean CAP snap bean diversity panel. Output from GAPIT using EMMA model with one PCA. Horizontal line represents the Bonferroni cut-off ($\alpha = 0.05$).
